# Research progress of diabetic retinopathy and gut microecology

**DOI:** 10.3389/fmicb.2023.1256878

**Published:** 2023-09-07

**Authors:** Rui Wang, Qiu-Yuan Wang, Yang Bai, Ye-Ge Bi, Shan-Jun Cai

**Affiliations:** ^1^Department of Ophthalmology, Affiliated Hospital of Zunyi Medical University, Zunyi, China; ^2^Special Key Laboratory of Ocular Diseases of Guizhou Province, Zunyi, China

**Keywords:** diabetic retinopathy, gut microbiota, gut microecology, gut-retinal axis, metabolite

## Abstract

According to the prediction of the International Diabetes Federation, global diabetes mellitus (DM) patients will reach 783.2 million in 2045. The increasing incidence of DM has led to a global epidemic of diabetic retinopathy (DR). DR is a common microvascular complication of DM, which has a significant impact on the vision of working-age people and is one of the main causes of blindness worldwide. Substantial research has highlighted that microangiopathy and chronic low-grade inflammation are widespread in the retina of DR. Meanwhile, with the introduction of the gut-retina axis, it has also been found that DR is associated with gut microecological disorders. The disordered structure of the GM and the destruction of the gut barrier result in the release of abnormal GM flora metabolites into the blood circulation. In addition, this process induced alterations in the expression of various cytokines and proteins, which further modulate the inflammatory microenvironment, vascular damage, oxidative stress, and immune levels within the retina. Such alterations led to the development of DR. In this review, we discuss the corresponding alterations in the structure of the GM flora and its metabolites in DR, with a more detailed focus on the mechanism of gut microecology in DR. Finally, we summarize the potential therapeutic approaches of DM/DR, mainly regulating the disturbed gut microecology to restore the homeostatic level, to provide a new perspective on the prevention, monitoring, and treatment of DR.

## 1. Introduction

With the improvement of people’s living standards and the accelerated pace of life, the incidence of diabetes mellitus (DM) is increasing year by year. The global prevalence of diabetes (20–79 years old) was 10.5% (536.6 million people) in 2021, and it is estimated to rise to 12.2% (783.2 million) by 2045. Global DM-related health spending is estimated at $ 966 billion in 2021 and is projected to reach $ 1.054 trillion by 2045 (Sun et al., 2022). Diabetic retinopathy (DR) is the most common microvascular complication caused by the continuous increase of blood glucose in the course of DM (Faselis et al., 2020). There are no subjective symptoms in the early stage, and varying degrees of visual impairment occurs with the development of the disease, which is the main cause of moderate to severe visual impairment and blindness worldwide (Jampol et al., 2020). Clinically, DR is divided into two stages: non-proliferative diabetic retinopathy (NPDR) and proliferative diabetic retinopathy (PDR). NPDR is an early manifestation of DR, characterized by retinal changes such as microaneurysm, hemorrhage, and exudation. PDR is the late stage of DR, characterized by neovascularization (Zhang et al., 2021). The prevalence of DR in the general population is only 1.14%, but it is as high as 18.45% in DM patients (Song et al., 2018). Therefore, it is an urgent problem for global ophthalmologists to explore the pathogenesis and new treatment methods of DR. Finding any way to prevent or delay the occurrence and development of DR will bring immeasurable practical significance to DR patients and the whole social and economic development.

The main pathological mechanism of DR is chronic low-grade inflammation and microangiopathy of the retina. The inflammatory cascade of the retina plays an important role in the development of DR. Continuous high glucose stimulates retinal cells, accompanied by a decrease in phagocytic capacity, leading to the accumulation of ATP, reactive oxygen species (ROS), mitochondrial DNA and other metabolites, inducing the activation of NLRP3 inflammasome, further increasing the expression of inflammatory cytokines (IL-18, IL-1β, ICAM-1) through the NF-κB pathway (Liu et al., 2018; Choi, 2023; Zheng et al., 2023). At this time, high levels of inflammation in the retina induce leukocyte deposition and adhesion, glial cell activation, pericyte loss, and endothelial cell overactivation (Kovoor et al., 2022). These changes further disrupt the blood-retinal barrier (BRB), promote overexpression of vascular endothelial growth factor (VEGF) and ROS, and ultimately lead to retinal oxidative stress, vascular leakage, occlusion, and even neovascularization (Kovoor et al., 2022; Zhang et al., 2022). In addition to the above traditional pathological mechanism of DR, the changes in gut microbiota (GM) flora structure and its metabolites have gradually attracted the attention of scholars, but the potential correlation mechanism between gut microecology and the progression of DR are still being investigated. This review summarizes the structure of the GM flora and the corresponding changes of its metabolites in DR, also expounds on the mechanism of intestinal microecology and DR. Moreover, the use of probiotics, supplementation of derivatives such as bile acid (BAs) and short-chain fatty acids (SCFAs), dietary changes, and fecal microbiota transplantation to regulate disordered gut microecology to restore homeostasis levels were discussed. The results further provide a basis for the potential pathways of gut microecology in the occurrence and development of diseases, which is of great significance and provides a new perspective for maintaining a healthy organism and the treatment or monitoring of diseases.

## 2. Gut-retinal axis

GM refers to the microorganisms that live in the human gastrointestinal tract and are in dynamic equilibrium with the organism. The structure of the GM flora and its metabolites in the organism constitute an organic environment, known as the gut microecology. At present, more than 1,000 species have been identified. The most abundant species are *Firmicutes* and *Bacteroidetes*, and others include *Proteobacteria*, *Actinobacteria*, *Fusobacteria, Verrucomicrobia*, etc. (Walsh et al., 2014). These floras are combined in proportion to maintain a relative balance at the level of abundance and diversity, thereby promoting the organism and the GM flora to be in a steady state. When the internal and external environment of the organism changes, a variety of pathogenic bacteria in the gut grow rapidly, while the beneficial bacteria are greatly reduced, the ability of the organism to resist changes in the environment is diminished, and the defense ability against harmful microorganisms such as pathogenic bacteria is weakened, which leads to a disruption of the GM flora.

In a variety of diets and drugs must enter the gastrointestinal tract and be digested, reabsorbed, produced, or excreted by the GM flora, and then play their respective roles. The metabolites produced by GM mainly include lipopolysaccharide (LPS), trimethylamine N-oxide (TMAO), BAs, and SCFAs. In recent years, a large number of studies have reported that there is a certain relationship between GM and eye diseases. Gut microecology disorders are closely associated with a variety of diseases such as DM (Tong et al., 2018), obesity (Zeng et al., 2019), inflammatory bowel disease (Cox et al., 2020), uveitis (Hu et al., 2021), age-related macular degeneration and choroidal neovascularization (Li et al., 2021). Subsequently, the hypothesis of gut-eye axis and gut-retinal axis was put forward (Kugadas et al., 2017; Rowan and Taylor, 2018; Floyd and Grant, 2020; Liu et al., 2022a). The leakage of the GM flora and its derived peptide peptidoglycan (PGN) can induce the inflammatory environment and blood-retinal barrier (BRB) decomposition in the eye. Gut-derived peptide PGN enters systemic blood circulation and activates Toll-like receptor 2(TLR2) on endothelial cells, leading to leakage of the retinal barrier (Prasad et al., 2022). This study reveals that the microbiota in the gut and plasma may affect the interior of the globe and retina by activating the TLR signaling pathway, due to the damage of the gut barrier and BRB in DR. This activation may create an environment for potential pathogenic microorganisms to be present in the retina which can lead to pathological changes in DR (refer to Table 1). This further provides relevant support for the gut-retinal axis hypothesis. However, exploring its specific mechanism still requires great challenges. GM may regulate the production of inflammatory cytokines by regulating an organic gut microecology composed of flora structure (flora level, abundance, diversity, similarity, etc.) and flora metabolites (LPS, Bas, TMAO, SCFAs, etc.), thereby improving the inflammatory environment of the retina and affecting its cell function to participate in the occurrence and development of DR. Although the research on the association between GM and DR is still in its initial stage, the in-depth investigation of the relationship between GM and DR can help to reveal the pathogenesis of DR and provide new perspectives on the treatment methods.

**Table 1:** The structural changes of the GM flora in DR and their associated effects.

Species	GM flora (In the DR group)	Effects and concerns
Human: DR/T2DM/HC India (Das et al., 2021)	*Bacteroidetes*, *Actinobacteria*↓ Anti-inflammatory: *Faecalibacterium*, *Roseburia*, *Blautia*, *Mitsuokella*↓ *Lachnospira*↓; *Akkermansia*, *Alistipes*↑ Probiotic/Anti-inflammatory: *Bifidobacterium*, *Lactobacillus, Streptococcus*↓ Pro-inflammatory: *Methanobrevibacter*↓; *Shigella*↑ Pathogen: *Desulfovibrio*, *Erwinia*↓; *Escherichia*, *Entercoccus*, *Enterobacter*, *Cloacibacillus*↑ Alpha/Beta diversity: Shannon↑/ Significant differences in GM flora	Altered balance between Anti-inflammatory and Pro- inflammatory GM flora.
Human: DR/DM/HC China (Huang et al., 2021)	*Firmicutes*↓; *Bacteroidetes*↑ *Faecalibacterium*, *Escherichia-shigella*, *Eubacterium-hallii*, *Clostridium*↓ *Bifidobacterium*, *Lactobacillus*, *Desulfobacterota*, *Eubacteriaceae*↑ LFfse analysis: DR-18 GM flora; DM-11 GM flora; HC-34 GM flora Common genus (211): DR-22 unique genus; DM-8 unique genus; HC-13 unique genus Alpha/Beta diversity: Invsimpson, Shannoneven, Heip↓ / Gut microecology dysbiosis	Pasteurellaceae: can be used as a potential biomarker to distinguish DR/DM.
Human: DR /HC China (Bai et al., 2022)	Phylum level: *Firmicutes*↓; *Bacteroidota*, *Synergistota*↑ *Desulfobacterota*, *Verrucomicrobiota*↑ Genus level: *Blautia*, *Eubacterium-hallii*, *Anaerostipes*↓ *Romboutsia*, *Collinsella*↓ *Bacteroides*, *Megamonas*, *Ruminococcus-torques*↑ *Alistipes*, *Escherichia-shigella*↑ LFfse analysis: DR-68 different GM taxa; HC-33 different GM taxa Common genus (196): DR-45 unique genus; HC-20 unique genus Alpha/Beta diversity: Sobs, Ace, Shannon↑/ HC-DR: obvious differences	Potential biomarkers: *Bacteroides*, *Megamonas*, *Blautia*, *Anaerostipes*, *Romboutsia* Insulin resistance↓ Glucosinolate biosynthesis↓
Human: PDR /NDR China (Ye et al., 2021)	Phylum level: F/B ratio (−) Family level: *Coriobacteriaceae*, *Veillonellaceae*, *Sterptococcaceae*↓; *Burkholderiaceae*↑ Genus level: *Morganella*↑ Alpha/Beta diversity: OUT, Chao1, Shannon, Simpson↓/ PDR-NDR: Apparent separation of GM flora between two groups GM flora and metabolite interactions promote PDR development	Amino acid metabolism↑ Lipid metabolism↑ Arachidonic acid metabolism, Microbial metabolism, Linoleic acid metabolism, Purine metabolism↑
Human: STDR/T2DM India	B/F ratio↑; Any GM flora abundance (−) Optimal cutoff for B/F ratio 1.05, with significant clustering of cases above this value in beta diversity	Potential biomarker: Increased B/F ratio (Khan et al., 2021)
Human: DR/DM/HC India (Das et al., 2023)	Microbiota in the aqueous humor: Anti-inflammatory: *Faecalibacterium*, *Roseburia*, *Lachnospiraceae*↓ *Prevotellaceae*, *Ruminococcus*, *Butyrivibrio*↓ Pathogen: *Haemophilus*↓; *Shuttleworthia*↑ Alpha/Beta diversity: OUT, Chao1↓/ DR-forms a unique cluster GM: DR/DM-Ecological dysbiosis	Dysbiosis of the microbial ecology of the aqueous humor is different from GM. Presence of intraocular microbiota in the eye.
Akita mice/WT: Fecal/Plasma Ocular globe /Retina (Prasad et al., 2022)	Fecal, Globe: F/B ratio↓; Plasma, Retina: F/B ratio↑ Fecal: *Akkermansia*, *Bifidobacterium*, *Lactobacillus*↓; *Bacteroides*↑ Plasma: *Akkermansia*, *Bifidobacterium*, *Lactobacillus*↓ *Faecalibacterium*, *Staphylococcus*, *Bacteroides*, *Clostridium*↓ *Corynebacterium*, *Propionibacterium*↑ Globe: *Akkermansia*, *Faecalibacterium*, *Staphylococcus*, *Corynebacterium*↓ *Bacteroides*, *Clostridium*, *Propionibacterium*, *Pseudomonas*↑ Retina: *Corynebacterium*, *Pseudomonas*↓ *Staphylococcus*, *Enterococcus*, *Bacillus*↑ Alpha/Beta diversity: (−) / Fecal↓; Plasma, Globe, Retina↑	Retina: PGN, TLR2↑ Retinal inflammation↑ Retinal function↓ Acellular capillaries↑
Mice: db/db db/m (Beli et al., 2018)	After intermittent fasting (IF) intervention: F/B ratio↑ *Akkermansia*, *Bifidobacterium*, *Verrucomicrobia*, *Allobaculum*, *Bacteroides*↓ *Ruminococcus*, *Oscillospira*, *Lactobacillus*↑ Alpha/Beta diversity: (−) / Apparent separation, DR: reorganized a unique GM	IF: acellular capillaries↓ Retinal inflammation↓ TUDCA, survival period↑
Human: T2DM/HC Colombia	After metformin treatment in T2DM patients: *Akkermansia*, *Bifidobacterium*↑ *Prevotella*, *Butyrivibrio*, *Megasphaera*↑	Metformin-associated with SCFAs-producing GM flora (de la Cuesta-Zuluaga et al., 2017)
Human: T2DM/HC China	After metformin treatment in T2DM patients: *Blautia*↑; *Akkermansia*↓; *Simpson*↑ After AMC treatment: *Faecalibacterium*, *Blautia*↑; Chao1↓	Insulin resistance↓ Plasma triglyceride↓ (Tong et al., 2018)

## 3. Mechanisms of gut microecology influencing the onset and development of DR

Gut microecology disorders are closely associated with DM and its complications, including the effects of an inflammatory response, metabolism, vascular permeability, oxidative stress, and insulin resistance (Qin et al., 2012; Arora and Bäckhed, 2016; Allin et al., 2018). Under the condition of continuous increase of blood glucose, the GM flora and its metabolites are unbalanced, and the barrier against pathogenic microorganisms is weakened, which increases the opportunity to destroy the metabolism and immune level of the organism, leading to changes in the entire micro-ecosystem, termed gut microecology disorders. The following section focuses on the two main aspects of the GM flora structural and flora metabolites that are altered in DR, as well as the mechanism of gut microecology targeting to regulate the retinal inflammatory microenvironment of this altered, ultimately how this affects the development of DR.

### 3.1. GM influences DR through the structure of the flora

The relative abundance ratio of *Bacteroidetes* to *Firmicutes* (B/F ratio) was associated with DR, the B/F ratio was increased in GM of DR patients (Khan et al., 2021). Based on this study, the increased B/F ratio of the GM in T2DM patients may be a potential biomarker for sight-threatening DR. DR patients showed ecological imbalance from the phylum to the genus level (Bai et al., 2022). At the phylum level, *Firmicutes* decreased, *Bacteroidota* and *Desulfbacterota* enriched in DR patients. At the genus level, *Bacteroidetes* are the most abundant. This is consistent with previous studies. A Mendelian random study showed that *Chritensenellaceae* and *Peptococcaceae* were protective factors for DR at the family level, and *Ruminococcaceae* and *Eubacterium* were associated with the risk of DR at the genus level (Liu et al., 2022b). We note that these floras belong to *Firmicutes* in this study. Consequently, this result potentially suggests that we should not only focus on major categories but also changes in specific floras when studying GM flora. Another study found that the *Pasteurellaceae* flora can be used as a potential marker to distinguish DR and DM (high accuracy AUC: 0.74) (Huang et al., 2021). Furthermore, whether this flora can be used clinically to monitor the development of DM/DR patients remains to be explored, but we expect that the potential biomarkers of these florae can be used as a powerful point for us to monitor the disease in the future.

There are significant differences in GM between DR patients and normal people (Das et al., 2021). Through 16SrRNA sequencing analysis, the gut microecology of DR patients has been disordered. In addition, the anti-inflammatory bacteria (*Roseburia*, *Anaerostipes*, *Coprococcus*, *Streptococcus*, *Phascolarctobacterium*, *Faeculibacterium*, *Ruminococcus*) and probiotics (*Bifidobacterium*, *Lactobacillus*) were found to be reduced. There was a decrease in the pro-inflammatory bacteria *Sutterella*, but an increase was observed in *Shigella*. The pathogenic bacteria such as *Haemophilus* and *Rothia* were reduced, but *Escherichia* and *Enterobacter* were greatly increased. The GM flora in the organism can regulate the inflammatory response by changing the levels of pro-inflammatory factors, anti-inflammatory factors, chemokines, and proteins. For example, the *Faecalibacterium* flora inhibits the activity of IL-6, IL-17, and HsCRP (van den Munckhof et al., 2018). *Lactobacillus* and *Bifidobacterium* are classified as beneficial bacteria, which can play a variety of health-promoting effects on the organism (Hill et al., 2014). They can reduce inflammation and islet β cell dysfunction by reducing the expression of cytokines IL-6, IL-1β, IL-8, and circulating LPS levels (Tian et al., 2016). *Escherichia* and *Enterobacter* produce misfolded amyloid proteins that affect the development of DM (Tetz et al., 2019). In summary, with the increase and decrease of the flora in these categories of anti-inflammatory, pro-inflammatory, and pathogenic, the gut microecological balance is disturbed, which promotes the disruption of the GM flora and induces an inflammatory response. This result is slightly different from the theoretical assumption of DR chronic inflammation, which further leads to the consideration of whether the internal imbalance of anti-inflammatory and pro-inflammatory flora is the cause of DR chronic inflammation.

**Figure 1:**
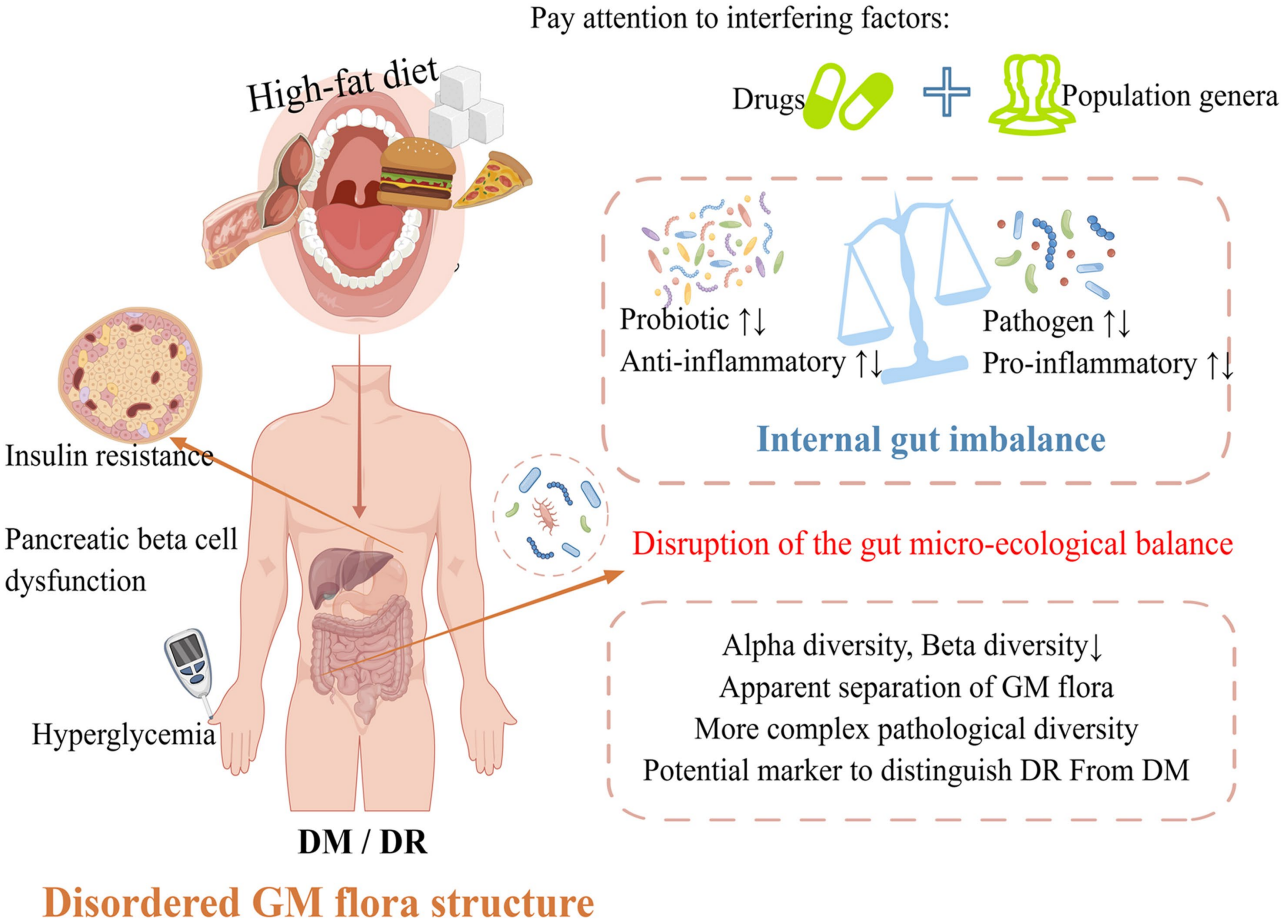
Changes in the structure of the GM flora in DM/DR patients.

The structure of GM flora is not only at the level of each phylum but also includes its diversity, similarity, and abundance. α-diversity refers to the GM biodiversity within the organism, including the richness index (Ace, Chao, OTUs), the diversity index (Shannon, Simpson, Invsimpson), and the evenness index (Heip, Shannoneven). β-diversity refers to the diversity differences among individuals, which are generally detected by UniFra, PERMANOVA, Anosim analysis and similarity analysis by UPGMA clustering after principal coordinate analysis (PCoA). DR patients with gut microecology disorders will show more complex pathological diversity (Huang et al., 2021). DR patients exhibited lower α-diversity and β-diversity in comparison to healthy individuals. Additionally, the probiotics *Bifidobacterium* and *Lactobacillus* showed an increase in abundance, as well as a decrease in the relative abundance of *Firmicutes*, and enrichment of *Bacteroidetes*, *Desulfbacterota*. This result is quite different from the above studies. The reason may be that some DR patients are affected by metformin or other drugs during the course of the disease. As studies have found that metformin and AMC (herbal formula) can significantly change the GM flora structure of T2DM patients (de la Cuesta-Zuluaga et al., 2017; Tong et al., 2018). In addition, compared with DM patients without DR, the α-diversity of PDR patients decreased (manifested as decreased OTUs, Chao 1, Shannon, Simpson), and the β-diversity based on PCoA showed a significant separation of the GM flora between the two groups (Ye et al., 2021). A study carried out in India demonstrated an increase in α-diversity in patients with DR (Das et al., 2023). This also further reminds us that in the study of the association between GM flora and DR/DM or other metabolic diseases, apart from looking at the effects of drugs, we should also pay attention to different species of populations (Figure 1; Table 1).

Several limiting factors must be taken into account when considering the aforementioned changes in the structure of the GM flora in DR. Firstly, the imbalance in the GM flora will be affected by some confounding factors such as race, age, gender, sample size, dietary habits, lifestyle, drugs, and fecal sampling methods. Secondly, on the one hand, most of the subjects were admitted to the hospital. There was some variation in the admission rate and selective bias. On the other hand, based on these cross-sectional studies, the causal relationship between the differences in the GM flora could not be precisely revealed. Finally, it is imperative to obtain additional evidence to confirm the restoration of the disordered gut microecology in disease treatment. Despite the various limitations of these studies, we also have a broad and preliminary understanding of gut microecology in DR. In the future, researchers should take these limitations fully into account in their research. The potential for bias can be reduced by using multi-center and large-sampled cohort studies. In addition, scientists should not limit themselves to clinical research, which should be supplemented by animal/cell experiments to improve the relevant mechanism of action, and further elucidate the causal relationship between GM flora and DR.

### 3.2. GM influences DR through the metabolites of the flora

**Figure 2:**
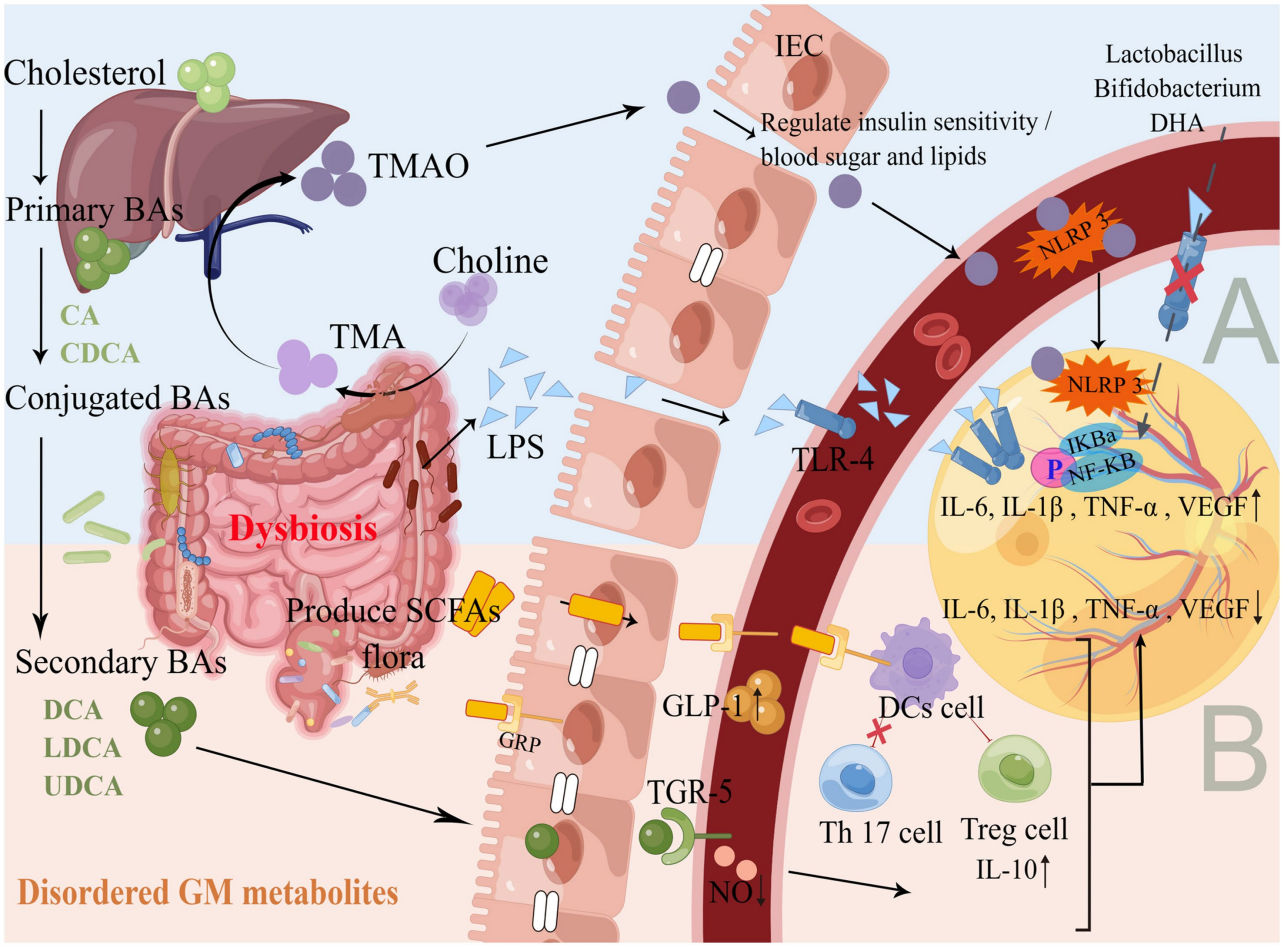
Production and mechanism of GM flora metabolites. **(A)** Promote inflammatory response. As shown in Figure 1, the GM flora of DR is disordered, and a large number of GM gram-negative bacteria accumulate to produce LPS. GM flora lyses choline and gradually oxidizes through the liver portal vein to produce TMAO, which binds to TLR4 and NLRP3 on the surface of blood vessels and the retina through IEC with impaired barrier function, activating the NF-κB pathway, up-regulates inflammatory cytokines and releases them into the retinal microenvironment. Probiotic supplementation and DHA diet can inhibit the above pathways to reduce inflammation and oxidative stress. **(B)** Inhibition of inflammatory response. The liver and GM flora gradually produce secondary BAs, *Faecalibacterium*/*Akkermansia*/ *Phascolarctobacterium* and other GM flora to produce SCFAs, which are absorbed through the gut and combined with TGR5 and GRP to maintain IEC homeostasis, promote the secretion of GLP-1 in the blood, reduce NO levels, induce DCs to activate Treg cells and inhibit Th17 cells, up-regulate anti-inflammatory factors and down-regulate pro-inflammatory factors to act synergistically on the retina to maintain gut and retinal homeostasis. Supplementation with BAs and SCFAs can improve the immune system, the metabolism, and the anti-inflammatory capacity of the organism.

GM flora produces metabolites such as lipopolysaccharide (LPS), trimethylamine oxide (TMAO), BAs, and SCFAs. These metabolites are absorbed by the colon and released into the blood through the portal vein, where they are further released into the peripheral organs, such as the eyes neovascularization (Koh et al., 2016; Schroeder and Bäckhed, 2016). This gut-blood-retinal axis circulatory process is also accompanied by the regulation of cytokines, proteins and other modifications, such as promoting or inhibiting the production of inflammatory factors, activating immune regulation, leading to chronic persistent low-level inflammatory response, thus participating in the development and progression of DR (Figure 2).

#### 3.2.1. LPS: a metabolite of GM flora associated with DR and immune-inflammatory responses

LPS is an important metabolite of gram-negative bacteria in the gut. The dysbiosis of the GM flora in hyperglycemia leads to an increase in gram-negative bacteria such as *Escherichia*, which in turn triggers the accumulation of LPS, a metabolite of GM, and disrupts the arrangement of the tight junction protein (Cldn), altering gut permeability and triggering the translocation of LPS into the circulation to cause metabolic endotoxemia (Xu et al., 2020; Liu X. et al., 2021). The expression of Toll-like receptor 4 (TLR4) and NF-κB is enhanced in the retina of DR, and the initial TLR-mediated inflammatory response is necessary for host defense and repair. Overexpression of TLR4 has been shown to be involved in the pathogenesis of retinal diseases through a dual mechanism (Alomar et al., 2021). On the one hand, they stimulate the release of inflammatory cytokines and regulate the immune response to retinal inflammation. On the other hand, they induce a retinal microenvironment conducive to oxidative damage.

Retinal M*ü*ller cell lesions play an important role in the development of DR. M*ü*ller cells are the major glial cell type in the retina, they are activated when the retinal microenvironment is disturbed by trauma, neurodegeneration, or other disease of the eye. LPS has the effect of endotoxin, as it enters the blood circulation and binds to TLR to induce NF-κB nuclear translocation, thereby promoting the release of inflammatory cytokines (IL-6, IL-1β, TNF-α) (Zeng et al., 2021). High levels of inflammatory cytokines act on the retinal microenvironment, which will cause the activation of M*ü*ller cells and aggravate the inflammatory response of the retina, even leading to microvascular occlusion and neovascularization, thereby promoting the development of DR (Hu et al., 2020; Li et al., 2020). GM flora *Lactobacillus*, *Bifidobacterium* can also inhibit the expression of inflammatory cytokines by inhibiting LPS-induced TLR4-linked NF-κB activation, and stimulating the secretion of IL-10, thereby reducing the inflammatory response (Rocha-Ramírez et al., 2017). In animal studies, retinal thinning and accelerated retinal endothelial cell damage occurred in hyperglycaemic mice due to systemic LPS exposure, thereby exacerbating the inflammatory microenvironment of the retina (Vagaja et al., 2013). LPS is a natural ligand of TLR, and TLR is an important factor in inducing signal cascade and activating the NF-κB pathway. Do GM flora and its metabolite LPS regulate the expression of various cytokines through this signaling pathway, which in turn regulates the occurrence and development of DR? To solve this problem, scientists still need to conduct a series of basic experimental studies to verify the mechanism of GM flora structure and LPS metabolite on the DR retinal inflammatory microenvironment. Although the causal relationship between them is not clear, the preliminary results seem to provide a potential target for future efforts.

#### 3.2.2. TMAO: a metabolite of GM flora associated with DR and immune-oxidative stress responses

TMAO and cardiovascular disease are the focus of current research. At the same time, TMAO promotes effector T cell activity, induces immune activation, and enhances the type I IFN pathway, which can act as a driver of antitumor immunity (Mirji et al., 2022). TMAO is produced by the GM flora that breaks down choline-containing TMA and then oxidized in the liver through the portal vein. *Proteobacteria*, *Clostridium*, *Desulfovibrionia*, *Escherichia* and Cut C genes are able to produce TMA, which further increases the level of circulating TMAO. Further, it is found that the increase in TMAO level is related to the higher risk of DR in DM patients (Liu W. et al., 2021). The level of TMAO in the blood of DR patients is increased and is closely related to the severity of DR. In addition, TMAO is not only elevated in the blood but also the aqueous humor of people with PDR, as subsequent studies have shown (Xue et al., 2023). Though there are some limitations in this study, such as no sequencing analysis of GM flora in patients, further exploration of combined metabolites, with specific attention given to TMAO. However, this provides a potential direction for our in-depth study of the gut-retina axis. The following points may help explain the effect of GM flora and its metabolite TMAO imbalance in DR, although there is no confirmed study on this direct link.

1. A multi-ethnic, cross-sectional study with a large sample size has shown that the association between TMAO and DM may be due to changes in the GM flora (Fu et al., 2020). *Lachnospiraceae*, *Prevotella*, *Desulfovibrio,* and other TMAO-related flora are associated with insulin resistance, then TMAO and its related GM flora may together promote an inflammatory response. In DR patients, it is worth noting that there is an enrichment of *Lachnospira* and *Prevotella* bacteria (Zhou et al., 2021). Associations exist between the GM flora and DR, but it appears that these associations are mediated by TMAO.
2. During weight loss intervention in obese patients, TMAO and choline regulate insulin sensitivity, glucose metabolism, and lipid metabolism (Heianza et al., 2019). By causing dysbiosis in the GM, obesity is known to worsen retinopathy and nephropathy (Li et al., 2022).
3. By activating inflammatory pathways, TMAO may further regulate oxidative stress and the inflammatory milieu. On the one hand, TMAO can activate the NLRP3 inflammasome, leading to the upregulation of IL-1β expression (Boini et al., 2017). This response can trigger inflammation in the endothelium. *In vitro* studies show that high glucose and TMAO have a combined effect that results in the overexpression of ROS and NLRP3 inflammasome in human retinal microvascular endothelial cells (HRMECs). This leads to increased retinal dysfunction and barrier failure (Xue et al., 2023). On the other hand, TMAO promotes NF-κB phosphorylation. Subsequently, it down-regulated anti-inflammatory factors in peripheral tissues and the central nervous system, up-regulated the expression of inflammatory factors (IL-6, TNF-α), and ultimately induced endothelial dysfunction and vascular inflammatory calcification (Seldin et al., 2016; Zhang et al., 2020).

When investigating the mechanism of action between gut microecology and DR in the future, TMAO may be a promising area to start with. However, a comprehensive evaluation of the potential relationships between the three factors should be considered.

#### 3.2.3. BAs: is a metabolite from GM flora linked to DR with a beneficial role?

BAs are decomposed from cholesterol in the liver through classical or alternative pathways into primary BAs (mainly cholic acid and chenodeoxycholic acid), and then converted into conjugated BAs through the bile duct duodenum into the gut, further metabolized by GM into secondary BAs (deoxycholic acid, ursodeoxycholic acid, lithocholic acid, etc.). 95% of BAs are reabsorbed in the ileum by binding to the apical sodium-dependent bile acid transporter and enter the liver through the portal vein. 5% of BAs in the colon are excreted with feces by GM, which is called “enterohepatic circulation” (Xiang et al., 2021).

BAs-activated receptors include farnesoid X receptor (FXR), takeda G protein-coupled receptor 5(TGR5)and vitamin D. BAs can bind to these receptors to alter the metabolic function and the inflammatory response of the organism. A study of intermittent fasting (IF) in DR mice showed that IF treatment increased the gut F/B ratio. The GM flora is reorganized to convert primary BAs into secondary BAs in blood circulation. The increased tauroursodeoxycholic acid (TUDCA)in the blood is reabsorbed and passed through the BRB, activating TGR5 in retinal ganglion cells, reducing the expression of inflammatory factors such as retinal TNF-α, and improving the function of retinal cells to protect the retina and delay DR (Beli et al., 2018). The integrity of BRB is essential for maintaining the normal function of the retina under physiological conditions. BRB rupture can lead to vascular leakage, even macular edema, and visual impairment, which is an early feature of DR (Wang and Lo, 2018). Ursodeoxycholic acid (UDCA) reversed the breakdown of BRB in the retina of DR mice and reduced NF-κB-mediated inflammatory pathways to inhibit retinal inflammation (Ouyang et al., 2018). In hyperglycemia and other retinopathy states, nitric oxide synthase (NOS) activity is spontaneously regulated and triggers superoxide production. Excessive superoxide will quickly react with NO and produce powerful oxidant peroxynitrite to induce protein inactivation, lipid peroxidation, and DNA damage, leading to cell death and tissue damage (Opatrilova et al., 2018). At the same time, the synthesis and release of NO induced by high glucose stimulation can interact with VEGF and cause retinal vascular disease. TUDCA plays a protective role in DR by reducing inflammation and protecting retinal blood vessels. For example, after TUDCA treatment in DR rats, the expression of ICAM-1, NOS, NF-ΚB P65, and VEGF in the retina can be significantly reduced, also serum NO content can be reduced. *In vitro*, studies have found that TUDCA can also reduce the proliferation of HRMEC induced by high glucose and reduce its NO content (Wang C. F. et al., 2016).

#### 3.2.4. SCFAs: is a metabolite from GM flora linked to DR with a beneficial role?

SCFAs are mainly produced by the GM glycolysis of carbohydrates such as polysaccharides. Recent studies have confirmed that SCFAs-producing gut microbiota include *Anaerostipes*, *Faecalibacterium*, *Akkermansia*, *Eubacterium*, *Roseburia*, *Phascolarctobacterium*, *Prevotella*, *Parasutterella*, *Lactobacillus*, *Bifidobacterium*, *Ruminococcus* (Koh et al., 2016). The above-mentioned studies on the structure of the GM flora structure showed that most of these SCFAs-producing flora showed a decreasing trend in DR. SCFAs play an important role in maintaining the health of the organism, which can stimulate the secretion of cytokines and regulate the immune system. In the long-term high glucose environment, the NF-κB pathway is activated, which can induce the phosphorylation of NF-κB p65 in HRMEC, thereby up-regulating the secretion of various inflammatory cytokines. The combination of SCFAs and G protein-coupled receptor (GRP) can induce the secretion of glucagon-like peptide-1 (GLP-1) (Yao et al., 2020), while GLP-1 can protect the organism’s islet β cells, improve insulin resistance, ultimately improve the symptoms of DM (Hwang et al., 2015; Wang et al., 2019). GLP-1 analog (Exendin-4) prevents BRB catabolism and inhibits NF-κB activation and expression of inflammatory cytokines (IL-6, IL-1β, TNF-α, ICAM-1), which ultimately improves the inflammatory internal environment of the retina to protect the retina of DR rats (Gonçalves et al., 2016). The study illustrates that SCFAs may traverse the BRB via systemic circulation, and consequently, reach the eye. This discovery provides further evidence to support the existence of the previously described gut-retinal axis (Chen et al., 2021). The butyrate in SCFAs can bind to GRP on the surface of DCs, which promotes Treg cell activation and inhibits TH 17 cell development. For instance, butyrate can inhibit the expression of Cldn-2 in the intestine, up-regulate IL-10 to repair retinal vascular abnormalities, and improve the inflammatory environment by down-regulating NF-κB activity, thereby inhibiting the occurrence and development of DR (Wang Y. et al., 2016; Zheng et al., 2017). It is worth noting that the *Desulfbacterota* flora can accelerate the degradation of butyrate through the β-oxidation pathway, leading to abnormal energy metabolism in the organism (Hao et al., 2020), while the flora in DR shows an enrichment trend.

## 4. The potential treatment for DR: regulation of the gut microecology

In summary, we summarized the structure of GM flora and the corresponding changes of its metabolites in DR, also expounded the mechanism of intestinal microecology and DR. Next, we focus on whether DR can be prevented or treated by adjusting the gut microecology. We started with the dietary changes and drug intervention, supplement of probiotics, the supplement of derivatives such as BAs and SCFAs, and Fecal microbiota transplantation (FMT) to provide different intervention strategies to regulate the intestinal microecology of DR disorders (Table 2).

**Table 2:** Effects of different interventional treatments on gut microecology.

Treatment	Alteration of gut microecology	Species/country
DHA/vegetables/ fruits/ fish/high fiber diet/ oleic acid.etc	TMAO-mediated PT3K-NF- κ B signaling pathway↓ (Shi et al., 2022); *Bifidobacterium*, *Lactobacillus*, *Akkermansia*↑; *Desulfovibrio*, *Klebsiella* and other pathogens↓; Glucose homeostasis↑ (Chen et al., 2023) Protective effect on DR (Shah et al., 2022)	Human: T2DM/DR mice: acute kidney injury
Acarbose	*Bifidobacterium*, *Lactobacillus*, *Eubacterium*↑ *Bacteroides*, *Blautia*, *Clostridium*↓ *Faecalibacterium*: positive/negative correlation with rice/bread *Akkermansia*: negative correlation with potato; The ratio of primary BAs/secondary BAs in plasma↑ LPS, MCP-1, PAI-1, FGF19↓	Human: T2DM China/Japan (Su et al., 2015; Gu et al., 2017; Takewaki et al., 2021)
Insulin+capsules containing three probiotic strains	*Bifidobacterium*, *Lactobacillus*, *Akkermansia*↑ HbA1c, Fasting blood glucose↓ Immune cytokine levels: IL-8, IL-17, TNF-α↓	Human: T1DM China (Wang et al., 2022)
Selective increase of Lactobacillus	The significant change in GM flora: SCFAs-producing flora↑ SCFAs、GLP-1↑	T2DM Rats (Qu et al., 2018)
Selective increase of *Akkermansia muciniphila*	Insulin sensitivity↑, body weight↓ Inflammation levels↓ Gut barrier function↑,LPS↓	Human: metabolic syndrome (Depommier et al., 2019)
Selective increase of Bifidobacterium	GLP-2↑; LPS↓ Disruption of tight junctions, gut permeability↓ Cytokine levels: IL-6, IL-1α, TNF-α↓	Obese mice (Cani et al., 2009)
TUDCA	TGR5↓ Retinal Inflammation, Vascular Permeability, ER Stress↓	DM-tie2-TNF mice (Lenin et al., 2023)
UDCA	Vascular integrity↑ Pericyte loss↓	DR mice (Chung et al., 2017)
SCFAs	Intraocular detection of SCFAS Inflammatory reaction: CD45+, IL-6↓ T cell activation ability↑	Uveitis mice (Chen et al., 2021)
Butyrate	Partial recovery of GM imbalance; ZO-1, Occludin↑ Enterococcus, Desulfovibrio, Escherichia-Shigella↑ Total SCFAs↑: Butyric acid, 4-Methylvaleric acid, Caproic acid Retinal thinning↓, activated microglial cells↓	DR mice (Huang et al., 2023)
FMT Exchanged GM-Young-Old-Aged mice	Donor age-specific GM flora↑ Disruption of epithelial barrier integrity↓ Retinal Inflammation↓: IL-1β, TNF-α, LBP, C3↓ RPE65↑; Lipid and vitamin metabolism pathways↑	Young (3 months)/ Old (18 months)/ Aged (24 months) mice (Parker et al., 2022)
FMT T2DM + metformin into mice (Sun et al., 2018)	*Bacteroides. fragilis*↓ TUDCA↑ FXR signaling↓ Impaired glucose tolerance, Insulin resistance↓	Human: T2DM Mice: transplant feces from individuals with T2DM + metformin to mice
FMT Healthy /Autologous donors to T1DM patients	Diversity of GM flora↑ Insulin production↑ Autologous FMT performed better than donor FMT	Human: T1DM (de Groot et al., 2021)
FMT + Lifestyle intervention Six healthy lean donors to T2DM	FMT: the abundance of GM flora↑ Butyric acid producing bacteria↑ Repeated FMT: the implantation of lean-related GM flora↑ FMT+ Lifestyle intervention: *Bifidobacterium*, *Lactobacillus*↑ Total cholesterol, low density lipoprotein↓	Human: obese patients with T2DM (Ng et al., 2022)

### 4.1. Dietary changes and drug intervention

Protective effects on DR from consumption of fruit, vegetables, fiber, fish, tea, oleic acid, and Mediterranean diet (Shah et al., 2022). For example, a high-fiber diet may enhance the serum metabolic level of T2DM patients by altering the GM flora (Chen et al., 2023). Docosahexaenoic acid (DHA) exists in most marine foods, such as fish, shrimp, and shellfish. It has a regulatory effect on the GM flora and immune function, such as the inhibition of neovascularization and ischaemic injury (Chiang et al., 2022). DHA-acidified Cur ester treatment can prevent the TMAO-mediated PT3K-NF-κB signaling pathway, thereby inhibiting inflammation, apoptosis, and oxidative stress (Shi et al., 2022). Therefore, a targeted diet may become a potential dietary pattern to prevent and treat the occurrence and development of related diseases, which still needs more research support.

At present, metformin, acarbose, and glipizide are the preferred drugs for T2DM. Regular medication can not only reduce the patient’s blood glucose but also play a protective role in microvascular complications to delay the development of DM. In recent years, studies have gradually found that these drugs can also improve metabolic function through the structure of GM flora and its metabolites. After acarbose treatment, *Bifidobacterium*, *Lactobacillus,* and other beneficial bacteria were enriched in T2DM patients, and the abundance of *Bacteroides*, *Clostridium,* and other bacteria decreased (Takewaki et al., 2021). It is worth noting that the study also found that these changes in flora may be closely related to habitual dietary intake. Furthermore, in addition to the above effects of GM flora, researchers have found that acarbose can improve insulin resistance by increasing the ratio of primary BAs/secondary BAs in plasma (Gu et al., 2017), and can also reduce the expression of cytokines such as LPS, prothrombin activator inhibitor-1 (PAI-1), monocyte chemoattractant protein-1 (MCP-1) in serum (Su et al., 2015). The effect of metformin on GM flora is not repeated here (see 3.1). In conclusion, these positive effects also suggest the importance of avoiding confounding factors during the clinical study of gut microecology in patients with DM/DR. In the future, the structure and metabolism of GM flora may provide a perspective when we choose drug strategies, as well as the intervention of dietary guidance may play a complementary role.

### 4.2. Supplement of probiotics

As outlined in Section 3.1, besides confounding factors like medication, most studies indicate reduced levels of probiotics, such as *Bifidobacterium* and *Lactobacillus*, in patients with DR. The intervention of probiotics and prebiotics is viewed as an effective supplement, which represents a new strategy with significant potential in the development of DM/DR. In a double-blind randomized controlled trial, patients with T1DM were treated with insulin and capsules containing three probiotic strains (Wang et al., 2022). The treatment increased the *Bifidobacterium*, *Lactobacillus salivarius*, and *Akkermansia muciniphila* flora in the body, which helped regulate immune cytokine levels, resulting in a more stable blood glucose level. This study focuses on the combined effect of multi-strain probiotic supplementation. However, individual effects of single probiotic strains, as shown in subsequent studies, need further consideration. T2DM rats were supplemented with the probiotic *Lactobacillus casei Q14*, which was derived from the fermented dairy product yak yogurt (Qu et al., 2018). The study found a significant change in the composition of GM flora, as well as selective enrichment of SCFAs-producing flora, leading to increased secretion of SCFAs and GLP-1 for the regulation of DM development. In contrast, in clinical trials of individuals with metabolic syndrome, who were administered daily oral doses of live or pasteurized *Akkermansia muciniphila* flora, it was observed that the overall structure of the GM remained unchanged (Depommier et al., 2019). Nevertheless, this study also found that the probiotics could improve gut barrier function, accompanied by a decrease in plasma LPS levels, further improving the host’s inflammatory response and insulinemia. Similarly, prebiotic supplementation in obese and DM mice resulted in improved inflammatory tension and gut barrier function. These effects were found to be altered by GLP-2-dependent mechanisms (Cani et al., 2009). It was also accompanied by increased abundance of the *Bifidobacterium* spp., *Lactobacillus* spp., and *Eubacterium rectale clusters*. Furthermore, research has demonstrated that probiotics could serve as a drug delivery vehicle for the management of DR in the future (Prasad et al., 2023). Although some positive results have been documented in these studies, issues related to the availability, formulation composition, and quality control of probiotic strains remain challenging. Due to the complex nature of GM flora and individual variations, we may also explore the possibility of personalized probiotic supplementation in the future.

### 4.3. Supplement of derivatives such as BAs and SCFAs

Could GM flora metabolites play an interventional role alongside the supplementation of effective strains? Based on the above 3.2 parts, we have learned that the GM flora and its metabolites were disturbed in the DM/DR. A logistic regression analysis suggests that BAs might function protectively, inhibiting the progression of T2DM into DR (Su et al., 2021). Consequently, BAs present a potential therapy target for retinal diseases. TUDCA is a metabolite of BAs with anti-inflammatory effects. Following treatment with TUDCA, DM mice were found to alleviate retinal inflammation, vascular permeability, and endoplasmic reticulum stress via the TGR5 receptor signaling pathway, ultimately resulting in improved visual function (Lenin et al., 2023). Ursodeoxycholic acid (UDCA) is a hydrolyzed form of TUDCA. Furthermore, the treatment of DR mice with UDCA leads to improved retinal vascular integrity and a reduction in pericyte loss (Chung et al., 2017).

Several previous studies have shown a decreasing trend in the levels of SCFAs and the abundance of the GM flora responsible for producing SCFAs during DR. Exogenous supplementation of SCFAs can improve the metabolism of amino acids, vitamins, and energy. Besides increasing the strains that can produce SCFAs, it is possible that supplementing with derivative SCFAs may also impact DR. On the one hand, SCFAs have the potential to decrease retinal inflammation by suppressing the NF-κB pathway, thereby restoring retinal homeostasis in the case of DR. On the other hand, the butyrate found in SCFAs can bind to the GRP43 receptors situated on the surface of intestinal epithelial cells (IECs), thereby repairing and maintaining their homeostasis (Fujiwara et al., 2018). This process prevents harmful products such as TMAO and LPS from entering the bloodstream. Meanwhile, supplementation of DR mice with butyrate not only increased the expression of tight junction proteins (including ZO-1 and occludin proteins) to restore gut barrier function but also further improved the thinning retina and visual function of DR (Huang et al., 2023). Interestingly, supplementing SCFAs *in vivo*/vitro reduced intraocular inflammation induced by LPS, which ultimately affected the function of retinal glial cells in the eye (Chen et al., 2021). This finding could potentially indicate that gut microecology is a complex and dynamic system. In summary, the majority of studies depend on a single metabolite, which restrains our comprehensive view of the dynamic relationship between disease and gut microecology. When studying gut microecology and DR in the future, it is necessary to comprehensively consider GM and its metabolites. Subsequently, the potential mechanism of action between GM and its metabolites should be further explored through basic experiments.

### 4.4. Fecal microbiota transplantation

The gut microecology and its host are in a dynamic equilibrium system. Perhaps we should focus on this whole system, rather than simply supplementing with effective strains and metabolic derivatives of GM flora. FMT plays a significant role in metabolic disorders, such as obesity and diabetes. Yet the evidence for DR and FMT is rather limited. Initially, in preclinical research: Mice that underwent FMT were fed in groups with obese and lean twins, based on the observed coprophagia in mice (Ridaura et al., 2013). It was found that the two affected each other to prevent weight gain and obesity. Obese mice that received FMT from metformin-treated T2DM patients showed changes in the abundance of GM and the expression level of the metabolic derivative TUDCA (Sun et al., 2018). This led to further improvement in glucose intolerance and insulin resistance by inhibiting the FXR signal transduction pathway. Secondly, in clinical research: FMT has been shown to prevent the decrease of insulin production in patients with T1DM, and no associated adverse events were reported during the follow-up period (de Groot et al., 2021). Simultaneously, the study revealed a close relationship between GM flora, its metabolites, and the functionality of residual β cells after FMT treatment. Moreover, the performance of autologous FMT was superior to that of donor FMT. Nonetheless, this study has certain limitations, including the lack of a placebo group for control purposes and no dietary intervention. The study design incorporated these limitations as part of a double-blind randomized controlled trial, which also showed that intervening in a patient’s lifestyle is exceedingly challenging (Ng et al., 2022). On the one hand, FMT treatment increased the abundance of GM in obese individuals with T2DM, particularly butyric acid-producing bacteria. Repeated FMT further improved the implantation of the gut flora. On the other hand, while combining FMT with lifestyle intervention might increase the relative abundance of *Bifidobacterium* and *Lactobacillus*, it would not affect the diversity of the GM flora. Finally, the gut-retinal axis has further promoted the research progress of FMT and DR. The expression of key cytokines associated with retinal inflammation can be reduced by transferring GM flora from young to old mice (Parker et al., 2022).

Despite the aforementioned limitations of FMT, it is necessary to consider the following aspects: (1) In animal experiments, antibiotics were generally used to treat participants before FMT was administered. The positive aspect was the ability to observe changes in the gut microecology of participants more objectively and clearly. Another consideration is whether this will lead to a bias in the research results. Furthermore, this method may result in adverse side effects for subjects, leading to ethical concerns and considerations for antibiotic management when applied in clinical studies. This enables us to concentrate on the delivery route of FMT, aiming to discover an implantation method that does not necessitate antibiotic pretreatment, among other options. More evidence is required. (2) Favorable tolerability in both the short and long term, and few adverse events, have been demonstrated in these studies. In order to prevent the spread of disease through FMT, the preparation of the treatment must follow strict standardization guidelines, and the selection of donors should undergo strict screening. (3) As with probiotics, the varying outcomes achieved through FMT may be associated with the particular composition, dosage, efficacy, and individual heterogeneity of the therapy. Moreover, variations in geography, ethnicity, dietary habits, and lifestyles may also contribute significantly. In summary, the mechanism behind the strategy of adjusting gut microecology in DM and its complications needs to be further clarified and proven clinically. Although faced with several challenges, the study of gut microecology is a comprehensive and dynamic exploration that has undeniably opened up a novel field of research.

## 5. Conclusion and outlook

In summary, GM is involved in the processes of nutrient absorption, energy metabolism, and inflammatory response, which is in a state of dynamic equilibrium with the organism. The structure of GM flora and its metabolites in the gut flow into various organs through blood circulation, such as releasing to the eye and entering the BRB, accompanied by changes in various cytokines and proteins, to improve the inflammatory microenvironment and vascular injury of the retina and regulating the immune level of the organism, ultimately affecting the occurrence and development of DR. Clinically, the treatment options for patients with DR mainly comprise anti-VEGF drugs, laser photocoagulation, and surgical interventions. Nevertheless, most patients receive treatment due to the presence of severe visual impairment. Based on this article, we hope that gut microecology may be a powerful approach to prevent the onset and development of DR, as well as to monitor changes in the disease, while also playing a role in the preclinical stage. With the rapid development of computer and medical technology, in the future, we may be able to use the changes in the structure of the GM flora and its metabolites to monitor or diagnose DR, more likely to prevent or treat DR by adjusting the GM flora or metabolites. For example, individualized treatment options such as ingesting a specific beneficial strain, supplementing derivatives such as BAs and SCFAs, changing dietary patterns, regulating a specific protein factor, or transplanting fecal microbiota. This also suggests that more researchers need to focus on the in-depth exploration of gut microecology and DR and other eye diseases in the future.

## Author contributions

RW: Writing – original draft, Writing – review & editing. Q-YW: Writing – review & editing. YB: Writing – review & editing. Y-GB: Writing – review & editing. S-JC: Conceptualization, Methodology, Writing – review & editing.

## Funding

The author(s) declare financial support was received for the research, authorship, and/or publication of this article. This research was supported by the Guizhou Province Science and Technology Support Program Project (Guizhou science and technology cooperation support [2023] general 265)”, and the “Guizhou Dendrobium Industry Development Research Center Project (Guizhou dendrobium cooperation 2019003)”.

## Conflict of interest

The authors declare that the research was conducted in the absence of any commercial or financial relationships that could be construed as a potential conflict of interest.

## Publisher’s note

All claims expressed in this article are solely those of the authors and do not necessarily represent those of their affiliated organizations, or those of the publisher, the editors and the reviewers. Any product that may be evaluated in this article, or claim that may be made by its manufacturer, is not guaranteed or endorsed by the publisher.
